# Key Guidelines for Responding to Reviewers

**DOI:** 10.12688/f1000research.154614.1

**Published:** 2024-08-13

**Authors:** Saida Hidouri, Hela Kamoun, Sana Salah, Anis Jellad, Helmi Ben Saad

**Affiliations:** 1 Department of Pediatric surgery, Zaghouan Hospital, Research Laboratory LR12SP13, University of Monastir Faculty de Medicine of Monastir, Monastir, Monastir, Tunisia; 2 Ibn Nafiss Pneumology Department, Abderrahmene Mami Hospital, Ariana, University of Tunis El Manar Faculty of Medicine of Tunis, Tunis, Tunis, Tunisia; 3 Department of Physical Medicine and Rehabilitation, Fattouma Bourguiba University Hospital Monastir, University of Monastir Faculty of Dental Medicine of Monastir, Monastir, Tunisia; 4 Hôpital Farhat HACHED, Laboratoire de recherche LR12SP09 «Insuffisance cardiaque», 4000 Sousse, Université de Sousse Faculté de Médecine de Sousse, Sousse, 4000, Tunisia

**Keywords:** Academic publishing, Manuscript evaluation, Manuscript review process, Peer assessment, Peer review, Research quality, Review, manuscript assessment, Reviewer comments, Revision guidelines

## Abstract

**Background:**

The process of preparing a scientific manuscript is intricate, encompassing several critical stages, including pre-writing, research development, drafting, peer review, editing, publication, dissemination, and access. Among these, the peer review process (PRP) stands out as a pivotal component requiring seamless collaboration among editors, reviewers, and authors. Reviewers play a crucial role in assessing the manuscript’s quality and providing constructive feedback, which authors must adeptly navigate to enhance their work and meet journal standards. This process can often appear daunting and time-consuming, as authors are required to address numerous comments and requested changes. Authors are encouraged to perceive reviewers as consultants rather than adversaries, viewing their critiques as opportunities for improvement rather than personal attacks.

**Methods:**

Opinion article.

**Aim:**

To equip authors with practical strategies for engaging effectively in the PRP and improving their publication acceptance rates.

**Results:**

Key guidelines include thoroughly understanding and prioritizing feedback, maintaining professionalism, and systematically addressing each comment. In cases of significant disagreement or misunderstanding, authors have the option to refer the issue to the editor. Crafting a well-organized and scientific “response to reviews” along with the revised manuscript can substantially increase the likelihood of acceptance. Best practices for writing an effective response to reviews include expressing gratitude, addressing major revisions first, seeking opinions from co-authors and colleagues, and adhering strictly to journal guidelines. Emphasizing the importance of planning responses, highlighting changes in the revised manuscript, and conducting a final review ensures all corrections are properly documented.

**Conclusion:**

By following these guidelines, authors can enhance their manuscripts’ quality, foster positive relationships with reviewers, and ultimately contribute to scholarly advancement.

## Introduction

The process of preparing a scientific manuscript involves eight fundamental steps: pre-writing, research development, drafting, editorial procedures including the peer review process (PRP), editing, publication, dissemination, and access. ^1^^–^^4^ A successful PRP typically entails collaboration between editors, reviewers, and authors. ^4^ Authors are responsible for conducting research, analyzing data, and writing the manuscript to present their findings clearly, accurately, and comprehensively. ^4^ Reviewers, on the other hand, evaluate the manuscript’s quality and provide ‘constructive’ feedback. ^4^ Their goal is to ensure that the research meets the journal’s standards and contributes significantly to the field. ^4^ Misunderstandings between authors and reviewers can lead to the frequent rejection of high-quality submissions. ^4^^,^^5^

Responding to reviewers’ critiques is one of the most stressful phases of the publication process. ^4^ Therefore, it is crucial to establish strategic methods for authors to handle reviewers’ feedback in alignment with ethical guidelines, ^6^ and to provide thorough protocols for addressing diverse reviewers’ comments and principles for implementing successful revisions. ^7^ Given that a document titled “Responses to Reviewers” is mandatory during the PRP, and recognizing that some authors may struggle with addressing reviewers’ comments, it is essential to develop authors’ skills in resubmitting research and clinical scholarship reports. ^8^ This is critical for the effective dissemination of the authors’ work.

This opinion article aimed to equip authors with the knowledge needed to engage effectively in the PRP and improve the chances of manuscript acceptance.

## Tips and advices

The PRP is a cornerstone of scientific publishing, ensuring the rigor and quality of research. ^4^ Authors must navigate this process skillfully to enhance their manuscripts and address reviewers’ feedback. ^4^ According to Day and Gastel, ^9^ “Responding to reviewers’ comments is not just about defending your work; it’s about engaging in a constructive dialogue to improve the manuscript”. To help authors defend their manuscript and engage in constructive dialogue. This paper emphasizes understanding critiques, prioritizing feedback, and maintaining professionalism throughout the process.

Table 1 summarizes the key guidelines and tips for responding to reviewers. The following sections describe these tips to help authors confidently address peer reviewers’ comments and move closer to publication.

**Table 1.** Key guidelines for responding to reviewers: a summary.

Tips	Guideline: Authors should	Key points: Authors should
Consume reviewers’ critiques	• Value feedback • Avoid personalizing critiques	• Regard feedback as opportunities for improvement • Address critiques systematically and patiently • Remain composed and evaluate each comment objectively
Read carefully and understand the reviewers’ or editor’s comments	• Thoroughly examine and categorize comments for clarity and understanding	• Break down comments into separate points • Distinguish between positive feedback and critiques • Address all points and number them accordingly
Look for an opinion	• Seek advice from co-authors and colleagues to gain different perspectives	• Consult with peers for feedback • Brainstorm with the research group • Reach consensus on addressing comments
Prioritize feedback dealing with requests for major revisions	• Focus on addressing major comments first, followed by minor ones	• Identify and prioritize major issues • Revise and clarify significant points • Address minor revisions for overall clarity
Be polite and respectful of all reviewers	• Maintain professionalism and gratitude towards reviewers	• Regard reviewers’ comments as constructive • Express gratitude • Avoid confrontational or defensive tones
Pay attention to details: Respond to every point raised	• Ensure all points raised by reviewers are addressed individually	• Be specific in responses • Include additional information if needed • Make minor corrections to improve clarity
Don’t argue every single comment	• Choose your battles; only refute suggestions with valid reasons	• Provide rational explanations for disagreements • Use evidence and references to support arguments
Ambiguous situations	• Handle unclear comments, conflicting feedback, and ethical concerns diligently	• Seek clarification for ambiguous comments • Address conflicting feedback with thorough examination • Report ethical concerns to the editor
Be thorough and plan responses	• Organize and systematically respond to each comment	• Provide detailed responses • Address each point individually • Ensure clarity and professionalism in explanations
Highlight changes in the revised manuscript	• Make it easy for reviewers to see changes in the manuscript	• Detail responses in a cover letter • Use typographical aids like different fonts or colors • Consider using a table format for clarity
Revise and improve	• Ensure all necessary corrections are made and changes are highlighted	• Highlight changes for easy verification • Follow the journal’s submission requirements • Check for errors in the final version
Review again	• Perform a final thorough review of the manuscript	• Ensure no errors due to changes • Consider using AI tools or expert translators for proofreading • Declare the use of AI tools if applicable
Express gratitude again	• Conclude with gratitude towards reviewers and the editor	• Acknowledge the efforts of reviewers • Demonstrate professionalism and courtesy • Reiterate thanks in the cover letter
Resubmit promptly	• Resubmit the manuscript quickly with detailed responses and highlighted changes	• Submit promptly to keep the work fresh in reviewers’ minds • Include a detailed cover letter and response table • Demonstrate commitment to improving the manuscript

## Consume reviewers’ critiques

Given that the review report is crucial for the editor’s publication decision, ^10^ authors should:

i) View feedback as a valuable opportunity to improve their work;
ii) Not perceive critical remarks from reviewers as personal attacks;
iii) Remember that reviewers are critiquing their work, not them as individuals ^10^;
iv) Approach negative or major comments with a neutral and objective perspective ^7^; leveraging them for improvement. Some frustrating or adverse comments may hold validity and significantly enhance the research, ^11^^–^^15^ and
v) Transcend feelings of frustration, sadness, and perceived unfairness. ^6^

It is crucial that authors recognize that while the article has not been rejected, the editor has given an opportunity for revision and resubmission. ^4^ To navigate this process effectively, the authors should advocate adhering to some general guiding principles, which are systematic review, patience and reflection, and maintain composure. First, authors should methodically examine comments, categorize them as “major” or “minor,” and store them securely. This structured approach ensures careful consideration and appropriate addressing of all feedback. Second, authors should let feedback sit for a couple of days before responding, and then avoid hastily formulating responses. ^7^ Authors are asked to “sleep on it” before starting the revision process. ^13^ Third, feeling overwhelmed by numerous revision suggestions is natural. However, authors should remain composed and evaluate each comment objectively. Rather than panicking, authors should approach revisions calmly and discern the value of each suggestion. ^13^

## Read carefully and understand the reviewers’ or editor’s comments/critiques/suggestions

After reviewing the comments, authors should:

i) Carefully examine the editor’s letter and consider all remarks provided by the reviewers. ^7^ The goal is to pinpoint the specific points emphasized and address any additional issues raised ^7^;
ii) Take sufficient time to understand each comment, ensuring they grasp the reviewers’ perspective and the precise issues highlighted. ^7^^,^^16^ If necessary, authors can break down comments into separate points ^7^^,^^16^;
iii) Distinguish between positive feedback and critiques or requests for revisions ^7^^,^^8^^,^^17^^,^^18^;
iv) Open the document where each reviewers’ comments are saved, number each comment, and label them accordingly, attributing each comment to the respective reviewer (e.g., Review 1, comment 1) ^6^; and
v) Evaluate whether the comments can be adequately addressed and whether the revisions align with reviewers’ expectations. ^16^

The objective is to show the editor and reviewers a commitment to the process by meticulously addressing each comment and implementing necessary changes.

## Seek opinion

The PRP can be complex, and addressing reviewers’ comments effectively requires careful consideration and expertise. ^4^ Consulting co-authors and colleagues well-versed in the work can provide valuable insights and perspectives that authors may not have considered on their own. ^15^ Brainstorming with the research group can generate innovative ideas, particularly when addressing complex reviewers’ criticisms. It also helps reach a consensus on how to address the comments. ^7^

## Prioritize feedback dealing with requests for major revisions

Reviewers typically structure their comments with a summary paragraph, followed by detailed constructive feedback and recommendations for refinement. ^10^ They categorize comments as “major” or “minor”.

Major comments reflect the most concerning issues that must be revised for the paper to be considered for publication. ^19^^,^^20^ These comments typically refer to the scientific and methodological aspects of a manuscript. ^4^ Effectively revising and clarifying major issues is fundamental to understanding the manuscript. ^16^^,^^21^

Minor issues, while important, do not typically influence the overall conclusions of the manuscript. These may include unclear statements, missing or incorrect references, unclear data presentation, grammatical concerns, and other minor clarifications. ^7^ Authors should make even minor requests and changes without engaging in disagreements with reviewers, even if they do not completely agree. ^22^

## Be polite and respectful of all reviewers/express gratitude

Throughout the process of responding to reviewers’ reports, authors should remember that reviewers are typically well-meaning colleagues who generously invest their time to assist authors in enhancing their study. ^4^ Therefore, it is essential to regard their comments as constructive feedback and express gratitude for their invaluable contributions. ^15^ Even if authors have reservations about a reviewer’s perceived understanding, it is not advisable to convey such impressions to the reviewer. ^13^ If the reviewers encounter difficulty comprehending certain aspects, it might be due to the authors’ failure to clarify the idea sufficiently. ^23^^,^^24^ Authors should remember that readers may vary in expertise, and some may be less experienced than the reviewers. ^15^ Thus, the authors should ensure that their work is clear and accessible to all readers, not just experts. ^23^ Therefore, if the reviewers do not understand certain points, it is appropriate to apologize for not making them clear and to strive to clarify them further. Proceeding with revisions demonstrates to the reviewers that authors take their comments seriously. ^8^

In some instances, authors may perceive reviewers’ criticisms as discourteous. ^17^ However, this could simply be a case of miscommunication. ^15^ Responding rudely is unwarranted, especially considering the ultimate goal of getting the manuscript published. ^23^^,^^25^ It is then strongly advised to maintain a polite tone throughout the response to the reviewers, and to refrain from emotional, confrontational, or defensive expressions, even if the authors disagree with the reviewers or feel that the requested changes are unnecessary. ^23^ Even if it appears that the reviewers are using the review as an opportunity to “teach” them, ^10^ it is important to remain composed and respectful and to welcome reviewers’ recommendations with a positive perspective. ^8^

## Pay attention to details: Respond to every point raised

Generally, reviews are bulleted. ^23^ If two distinct issues are brought up within one bullet point, authors should ensure that both critiques are explicitly responded to. ^23^ Interspersing responses, breaking up one bullet point with multiple answers, helps maintain clarity and ensures each criticism receives a thorough response. ^23^ If the authors are unable to respond to all points, it is reasonable to:

i) Be specific in the response and address all points raised ^13^;
ii) Avoid dodging difficult points by ignoring them;
iii) Include additional information; provide requested data or figures that were not in the manuscript if they support the argument;
iv) Add or update references and offer a supplementary file if the journal restricts the length of additional data ^13^;
v) Delete unnecessary material, including figures or tables;
vi) Shorten sections of the manuscript if necessary, specifying the extent of the reduction in words or percentage; and
vii) Make minor corrections to the text, such as or revising grammar and typographical mistakes. ^13^^,^^26^

This comprehensive approach demonstrates the authors’ commitment and consideration of the review to both the editor and the reviewers, facilitating a smoother re-evaluation of their manuscript. ^6^^,^^27^

## Be thorough and plan responses

To enhance the quality of their responses and maintain clarity and professionalism, authors should:

i) Organize responses systematically, addressing each comment with care and politeness, even if they disagree with the reviewers ^6^;
ii) Number the reviewers’ points and respond to them sequentially ^18^;
iii) Provide specific responses and address all points raised by each reviewer in a formal manner ^13^^,^^23^;
iv) Ensure that responses are detailed to facilitate better understanding by editors and reviewers;
v) Clearly explain each reviewer’s comments, respond to each criticism individually, point by point, and do not miss any comment ^4^^,^^7^^,^^8^^,^^15^;
vi) Offer clear and concise explanations of revisions where necessary ^4^;
vii) Submit responses directly below the reviewers’ comments, tightening language, correcting any grammatical mistakes or misspellings, and incorporating corresponding changes in the revised manuscript ^28^;
viii) Start each response to each comment with a clear and direct answer to the specific issue raised, providing a “yes” or “no” answer whenever possible. ^13^^,^^23^ The objective is to demonstrate to the reviewers that authors have taken their comments seriously and to convey the actions they have taken in response to their critiques promptly ^23^; and
ix) Comply, whenever feasible, with the requests of the reviewers. ^23^ Change and modify where it makes sense.

If authors believe that a particular comment falls outside the scope of their study, they should explain and clarify the reasons for their stance in cases where they disagree with the reviewers or feel that an additional experiment or analysis is unnecessary. ^13^

## Avoid arguing every single comment

Authors have the right to engage in discussions regarding reviewers’ comments, but it is best to avoid disputing multiple comments. ^29^ If encountering serious disagreement with reviewers, particularly if a suggestion is deemed unreasonable or requests excessive work that strays from the study’s objectives, authors can refute the suggestion and make no changes. ^7^ In such instances, it is imperative to:

i) Acknowledge the comment;
ii) Provide a concise, factual explanation justifying the decision not to implement the suggestion ^29^; and
iii) Justify any refusals in the response letter and in the comments. ^7^^,^^13^^,^^30^^,^^31^

It is worth noting that reviewers may only tolerate one instance of refusal. ^32^ Engaging in a “fight” with the reviewers is unwise, even if the suggestions are flawed or misunderstood. ^23^ Arguing extensively with the reviewers may frustrate them and jeopardize the opportunity to publish the manuscript. If a point of contention is substantial enough to jeopardize the manuscript’s integrity or the arguments presented, authors may appeal to the editor for intervention. ^23^

## Ambiguous situations

Sometimes, authors may encounter ambiguous or conflicting feedback, conflict of interest concerns, or suggested additions that exceed the journal guidelines. ^4^ In such situations, authors should handle reviewers’ comments with careful consideration and diligence. At times, authors may encounter unclear or ambiguous comments. In such situations, authors should seek clarification rather than provide an incorrect response. ^4^ Clarification can be requested in the cover letter or by contacting the editor via email. Sometimes, authors might receive conflicting feedback from two different reviewers. ^7^ In such instances, it is important not to become overwhelmed and to start by thoroughly examining both sets of comments, choosing the one that best matches the authors’ vision for the manuscript. ^30^ Authors should then address this selected feedback by making the required adjustments to their manuscript. Additionally, it is advisable to outline to the editor the two contradictory comments and provide arguments in support of the comment selected by the authors. ^7^^,^^32^ This serves to clarify the authors’ rationale behind the decision and helps ensure consistency and coherence in the revision process. In certain instances, suspicions of ethics violations may arise. For instance, a reviewer might reject a manuscript only to later conduct a similar study and publish it. ^32^ Similarly, a reviewer may recommend citing an article that does not align with the study’s scope. ^32^ In such cases, authors may choose to send a separate letter/Email to the editor to clarify the circumstances and possibly suggest a change of reviewer or a reconsideration of the comment. ^23^ This step is crucial for maintaining the integrity of the PRP and ensuring fair and ethical treatment of manuscripts.

## Reviewers’ requests and journal’s guidelines

If reviewers request additions such as references, tables, or text that exceed the journal’s limits, authors may encounter a dilemma. If they agree to these changes and believe they enhance the manuscript, authors should implement the changes and address the issue in their responses, seeking the editor’s authorization. ^7^ However, if authors find that these changes contradict their vision for the manuscript, they can use the recommendations for authors to justify refusing the change. Authors should then write to the editor to explain why they cannot comply with the reviewers’ request. ^23^^,^^30^ This approach ensures that the manuscript remains aligned with the author’s intentions while also respecting the journal’s guidelines and maintaining transparency in the revision process. ^23^^,^^30^

## Highlight changes in the revised manuscript - Use typography to assist the reviewers in navigating through responses

For the benefit of both reviewers and editors, authors should streamline the revision process and make it as easy as possible for them to navigate. Well-organized responses can reduce confusion and frustration, ultimately increasing the likelihood of acceptance. Here are some recommended practices:

✓ Detail the responses, highlighting significant changes in the revised version, such as new experiments or analyses that modify conclusions. ^13^ This allows the editor to easily track modifications without searching extensively in the revised manuscript;
✓ Address the response directly to the editor, rather than the reviewers. For example, use phrases like “We agree with the reviewer…” instead of “We agree with you …. »
✓ Ensure that every comment is addressed in consecutive order, aligning with the sequence of comments provided by the reviewers. One approach is to copy and paste all comments from reviewers and editors, inserting the response to each point directly below it in a distinct color or font to distinguish between comments and responses. ^15^^,^^16^

If the journal requires identifying changes on a single copy of the manuscript, authors may use Microsoft Word’s track changes feature or highlight with a marker. They may use different colored fonts or highlights to separate corresponding responses to different reviewers and clarify this in the cover letter. ^7^ However, authors should be mindful that this approach might result in a lengthy manuscript that is challenging to read and may not effectively juxtapose the reviewers’ comments with authors” changes.

Another effective approach to organizing responses to reviewers is to utilize a table format that includes the reviewers’ comments, author responses, and corresponding changes with page and line references in separate columns ^6^^,^^7^^,^^13^^,^^23^^,^^29^^,^^32^^,^^33^ (Appendix 1). ^34^

## Revise and improve

After addressing all comments, authors must ensure any necessary corrections are made to the manuscript. ^29^ It is crucial to meticulously check that all changes made are properly documented. Highlighting the changes is preferable as it makes them easier for reviewers and the editor to verify. ^4^^,^^30^ If the journal requires two versions of the revised manuscript - one with highlighted changes and another in a clean format - authors should comply. ^7^^,^^32^ It is important to carefully review the journal’s submission requirements for the revised version and adhere to the provided instructions, as they specify how changes should be incorporated. ^7^

## Review again

If English is not the authors’ native language, they should consider having the final version corrected by an expert translator. ^6^ Alternatively, authors can use Artificial Intelligence tools to assist with the proofreading process, but it is important to declare the use of such tools. ^3^ Before finalizing the manuscript, it is crucial to perform a final thorough review to ensure that no errors have arisen due to the changes made. ^7^

## Express gratitude again

Taking a moment to express gratitude towards the reviewers and the editor demonstrates professionalism and courtesy. Therefore, authors should not forget to reiterate their thanks to them to conclude their cover letter. ^4^^,^^23^^,^^30^

In practice, authors are asked to include an introduction and conclusion in their response letter. This provides a comprehensive and respectful response, enhancing the overall impression of the manuscript revision process. Authors should begin the response letter with a brief introductory paragraph expressing appreciation to the reviewers for their time and valuable feedback ^6^ (Appendix 1). ^34^ This introduction sets a positive tone and acknowledges the reviewers’ contributions. For example: “The authors sincerely thank the reviewers for their thorough and insightful feedback on our manuscript. We have carefully considered each comment and made the necessary revisions to address their concerns. We believe these changes have significantly improved the manuscript.” After addressing each comment, the authors should conclude the response letter with a closing paragraph summarizing the major changes made and reiterating gratitude for the reviewers’ input ^23^ (Appendix 1). ^34^ For example, “In conclusion, we have made substantial revisions to the manuscript based on the reviewers’ comments. We believe these changes have strengthened the quality and clarity of our work. We are grateful for the reviewers’ constructive feedback and look forward to your favorable consideration of our revised manuscript.”

## Resubmit promptly

It is advisable to resubmit the manuscript as quickly as possible, while the reviewers and the editor still have the work fresh in their minds. ^4^ A prompt resubmission, accompanied by a detailed response letter, a clear response table, and highlighted changes, can significantly increase the likelihood of paper acceptance. ^30^ By providing a comprehensive and organized submission, authors demonstrate their commitment to addressing reviewers’ feedback and improving the quality of the manuscript, thereby enhancing its chances of acceptance.

## Conclusion

The PRP serves dual purposes. ^32^ For publishers, it offers a comprehensive evaluation of manuscripts from diverse perspectives, guarding against potential pitfalls like plagiarism. ^32^ For authors, it offers invaluable external insights, enabling them to refine and enhance their work, thereby improving its prospects for acceptance. ^32^

Navigating reviewers’ critiques can be daunting for authors. ^23^ However, the reviewers’ comments are not personal attacks, but rather constructive contributions aimed at facilitating publication. ^23^ Handling reviewers’ feedback appropriately and ethically significantly enhances the likelihood of manuscript acceptance. ^23^ Therefore, authors should approach the PRP with diligence, carefully considering each comment and making necessary adjustments to strengthen their manuscript. Additionally, maintaining clear and respectful communication with reviewers and editors throughout the process fosters a collaborative and productive environment conducive to scholarly advancement.

**Take home message**

Responding to reviewers' comments effectively is crucial for the successful publication of a scientific paper. By following structured guidelines, authors can address critiques thoroughly and respectfully, leading to improved manuscripts and fostering positive relationships with reviewers.

## Ethical approval

The study design was determined to be exempt from human subjects’ research review, and therefore, formal approval was not required.

## Informed consent

No need.

## Data Availability

No data associated with this article. Zenodo: Appendix 1: Table format to respond to reviewers. DOI: https://doi.org/10.5281/zenodo.12819228. ^34^ The project contains the following extended data:
• [Appendix 1: Table format to respond to reviewers]. ^34^ Data are available under the terms of the Creative Commons Attribution 4.0 International license (CC-BY 4.0).
