# Supplementary material for: Key Guidelines for Responding to Reviewers
Source: F1000Res. 2024 Sep 20;13:921. Originally published 2024 Aug 13. [Version 3] doi: 10.12688/f1000research.154614.3 (PMC11377928; doi:10.12688/f1000research.154614.3)
Supplement: Peer review history

# Reviewer report: Reviewer response for version 2

**DOI:** 10.5256/f1000research.171546.r323126

**Published:** 2024-09-16

**Authors:** Meghit Boumediene Khaled

**Affiliations:** 1 Djillali Liabès University, Sidi-Bel-Abbès, Algeria

To the Editor,

 Thank you for the opportunity to review the revised article and the author responses. I have no further comments to add, and I am satisfied with the revisions made. I accept the article for indexing.

 Regards

Is the topic of the opinion article discussed accurately in the context of the current literature?

Yes

Are arguments sufficiently supported by evidence from the published literature?

Yes

Are all factual statements correct and adequately supported by citations?

Yes

Are the conclusions drawn balanced and justified on the basis of the presented arguments?

Yes

Reviewer Expertise:

Nutrition and Food science.

I confirm that I have read this submission and believe that I have an appropriate level of expertise to confirm that it is of an acceptable scientific standard.

# Response

**Published:** 2024-09-16

**Authors:** Helmi BEN SAAD

**Affiliations:** Université de Sousse, Tunisia

Thank you very much.

# Reviewer report: Reviewer response for version 1

**DOI:** 10.5256/f1000research.169662.r316507

**Published:** 2024-09-12

**Authors:** Meghit Boumediene Khaled

**Affiliations:** 1 Djillali Liabès University, Sidi-Bel-Abbès, Algeria

This is an interesting article that provides an insightful overview of the Peer Review Process (PRP), emphasizing its critical role in ensuring research quality and rigor. The manuscript highlights the dual benefits of PRP for both publishers, who rely on it to maintain high standards, and for authors, who receive valuable feedback to refine their work.

 The authors accurately address the significance of responding to reviewers’ critiques, which is indeed one of the most challenging parts of the publication process.

 Indeed, responding to reviewers can be stressful, as it involves addressing critiques that may challenge the core of the research. 

 In section " **Tips and advices**" correct and put advice instead of advices (uncountable noun)

 Overall, the tips provided are practical and aligned with the goal of enhancing the manuscript through the peer review process. It would be beneficial to expand on specific strategies for prioritizing feedback and provide examples of how to maintain professionalism when responding to particularly challenging critiques.

**Consume reviewers’ critiques**

 Additional tip:

 - **Seeking guidance:** If an author is unsure how to address a particular comment, they can reach out to the editor or a mentor for guidance.

**Seek opinion .....**

 Overall, this section provides a comprehensive overview of the strategies authors should employ when responding to reviewer comments.

 The text could benefit from incorporating specific examples of major and minor reviewer comments to illustrate the concepts more concretely.

**Ambiguous situations**

 in this paragraph the authors provided solid and actionable advice for authors dealing with ambiguous situations during the peer review process with important guidance on handling ambiguous situations that authors may face during the PRP.

 However, in **Ethical consideration**: this section could be strengthened by referencing specific guidelines or policies that journals or organizations like COPE (Committee on Publication Ethics) have in place to handle such situations. This would give authors a clearer path to follow.

**Reviewers’ requests and journal’s guidelines**

 This part offers a well-rounded approach to handling reviewers' requests that may conflict with journal guidelines, as well as practical advice on organizing and presenting revisions. 

 Moreover, it provides valuable, practical advice for authors dealing with the peer review process. It balances the need to respect both reviewers' suggestions and the authors' original vision, while also emphasizing the importance of organization and clarity in presenting revisions. Some improvements could be made by simplifying certain recommendations, providing clearer guidance on seeking editorial approval, and streamlining references.

 The suggestion to use tables to organize responses to reviewers is excellent. This method is often recommended as it allows for a clear, organized presentation of how each comment was addressed, helping both reviewers and editors to easily follow the changes made.

**Revise and improve**

 A solid foundation for authors navigating the manuscript revision process is offered in this section. It covers key areas such as documentation, communication, and final review, with a clear emphasis on professionalism and attention to detail.

 However, I recommend reducing repetitive focus on gratitude (A single, well-crafted expression of thanks may be sufficient). The inclusion of a brief discussion on the limitations of **AI tools** and a more detailed reference to the appendix would also improve the section’s overall effectiveness.

**AI Tool Use:** The mention of AI tools for proofreading is timely, but the section could benefit from a brief discussion on the limitations of these tools. Authors should be cautious, as AI tools may not catch all linguistic nuances or contextual errors. A recommendation to use AI tools as a supplementary measure, rather than a primary one, would be more balanced.

**Conclusion**

 The authors provide a strong summary of the PRP’s significance for both publishers and authors. The conclusion effectively encapsulates the dual roles of the PRP for both publishers and authors. By highlighting how the PRP not only serves as a quality control mechanism for publishers but also provides authors with valuable feedback, the conclusion underlines the significance of this process in academic publishing.

 I recommend the indexing of this paper after considering the above mentioned comments.

Is the topic of the opinion article discussed accurately in the context of the current literature?

Yes

Are arguments sufficiently supported by evidence from the published literature?

Yes

Are all factual statements correct and adequately supported by citations?

Yes

Are the conclusions drawn balanced and justified on the basis of the presented arguments?

Yes

Reviewer Expertise:

Nutrition and Food science.

I confirm that I have read this submission and believe that I have an appropriate level of expertise to confirm that it is of an acceptable scientific standard, however I have significant reservations, as outlined above.

# Response

**Published:** 2024-09-15

**Authors:** Helmi BEN SAAD

**Affiliations:** Université de Sousse, Tunisia

The authors sincerely thank the reviewer for his thorough and insightful feedback on our manuscript. We have carefully considered each comment and made the necessary revisions to address his concerns. We believe these changes have significantly improved the manuscript.

 Below are our specific responses to the reviewer positive comments/suggestions.

**COMMENT 1.**

 This is an interesting article that provides an insightful overview of the Peer Review Process (PRP), emphasizing its critical role in ensuring research quality and rigor. The manuscript highlights the dual benefits of PRP for both publishers, who rely on it to maintain high standards, and for authors, who receive valuable feedback to refine their work. The authors accurately address the significance of responding to reviewers’ critiques, which is indeed one of the most challenging parts of the publication process. Indeed, responding to reviewers can be stressful, as it involves addressing critiques that may challenge the core of the research.

**RESPONSE**

 We thank the reviewer for his thoughtful and encouraging feedback. We are pleased that you found our article to provide an insightful overview of the PRP and its essential role in maintaining research quality. We fully agree that responding to reviewers' critiques is one of the most challenging yet vital aspects of the publication process. Our aim was to offer guidance that could ease the stress associated with this task, and your recognition of our efforts is greatly appreciated. No action is needed at this stage.

**COMMENT 2.**

 In section "Tips and advices" correct and put advice instead of advices (uncountable noun).

 Overall, the tips provided are practical and aligned with the goal of enhancing the manuscript through the peer review process. It would be beneficial to expand on specific strategies for prioritizing feedback and provide examples of how to maintain professionalism when responding to particularly challenging critiques.

**RESPONSE**

 We thank the reviewer for his positive feedback and valuable suggestion.

 First, we have changed advices by advice.

 Second, we have expanded the section on prioritizing feedback and maintaining professionalism in the revised manuscript. Specifically, we included strategies such as:

 Prioritizing feedback: We have advised authors to categorize reviewer comments into major and minor critiques, focusing on major revisions that directly impact the study’s methodology, results, or conclusions. This approach helps authors address the most critical issues first. For example, if a reviewer requests a clarification on data analysis or the inclusion of additional experiments, these points should be addressed before responding to minor grammatical corrections or stylistic suggestions.

 Maintaining professionalism: We emphasized the importance of remaining objective and not personalizing critiques. We recommend authors carefully read the reviewer’s comments, take a moment to reflect, and respond thoughtfully, especially to challenging critiques. For example, if a reviewer’s tone seems harsh or overly critical, authors can acknowledge the reviewer’s input, express gratitude, and focus on the content of the critique without addressing the tone directly. For instance, a response might begin with, “We thank the reviewer for their insightful feedback on our methodology,” followed by a detailed explanation of how the comments were addressed or respectfully refuted with supporting evidence.

 We have added the following sentence in the section “Prioritize feedback dealing with requests for major revisions”:

 In practice, authors are advised to categorize each reviewer comments into major and minor ones, focusing on major revisions that directly influence the study’s methodology, results, or conclusions. This approach helps authors address the most critical issues first. For example, if a reviewer requests a clarification on data analysis or the inclusion of additional experiments, these points should be addressed before responding to minor grammatical corrections or stylistic suggestions.

 We have also added the following sentence in the section “Be polite and respectful of all reviewers/express gratitude”:

 In practice, authors are advised to remain objective and not personalizing challenging critiques.

**COMMENT 3.**

 Consume reviewers’ critiques

 Additional tip:- Seeking guidance: If an author is unsure how to address a particular comment, they can reach out to the editor or a mentor for guidance.

**RESPONSE**

 We thank the reviewer for this thoughtful suggestion.

 We have incorporated the additional tip on seeking guidance in the "Seek opinion" section. Specifically, we highlighted that if authors are unsure about how to address a particular comment, they can seek advice from either the editor or a mentor. This ensures authors are equipped to respond to all comments effectively.

 We have added the following sentence in the "Seek opinion" section:

 In practice, if authors are uncertain about how to address specific comments, they are encouraged to consult the editor in chief or reach out to a mentor with expertise in the field. This approach provides clarity and helps ensure that responses are well informed and appropriate.

**COMMENT 4.**

 Seek opinion .....

 Overall, this section provides a comprehensive overview of the strategies authors should employ when responding to reviewer comments.

 The text could benefit from incorporating specific examples of major and minor reviewer comments to illustrate the concepts more concretely.

**RESPONSE**

 We thank the reviewer for this thoughtful suggestion.

 We have added the following sentences inside the section “Prioritize feedback dealing with requests for major revisions”

 Examples of major comments include modifying the central hypothesis, the main algorithm, and redoing an experiment ( *e.g.*; the central hypothesis of the study needs to be revised to better align with the presented data. Specifically, the hypothesis should address the potential impact of variable X on outcome Y, which is currently not considered).

 Minor remarks may include unclear statements, missing or incorrect references, unclear data presentation, grammatical concerns, reformulation, adding additional references, adding an extra paragraph/table/figure, and adding an appendix ( *e.g.*; the introduction section would benefit from the inclusion of recent references on the topic, particularly those published in the last five years). ^7^

**COMMENT 5.**

 Ambiguous situations

 in this paragraph the authors provided solid and actionable advice for authors dealing with ambiguous situations during the peer review process with important guidance on handling ambiguous situations that authors may face during the PRP.

 However, in Ethical consideration: this section could be strengthened by referencing specific guidelines or policies that journals or organizations like COPE (Committee on Publication Ethics) have in place to handle such situations. This would give authors a clearer path to follow.

**RESPONSE**

 We thank the reviewer for this thoughtful suggestion.

 We have added the following sentences inside the section “Ambiguous situations”

 Finally, in case authors suspect that a reviewer has used artificial intelligence -assisted technologies to produce a review report (without acknowledging it), they need to alert the editors. **^36^** To handle such situations, authors can refer to specific guidelines and policies provided by international organizations (e.g., Committee on Publication Ethics, **^37^** International Committee of Medical Journal Editors **^38^**). These guidelines provides comprehensive resources and guidelines for ethical practices in publication, including how to handle conflicts of interest and other ethical issues. **^37, 38 ^** By following these guidelines, **^37, 38 ^** authors can ensure that they handle ambiguous and ethical situations appropriately, maintaining the integrity of the PRP and their research.

**COMMENT 6.**

 Reviewers’ requests and journal’s guidelines

 This part offers a well-rounded approach to handling reviewers' requests that may conflict with journal guidelines, as well as practical advice on organizing and presenting revisions. Moreover, it provides valuable, practical advice for authors dealing with the peer review process. It balances the need to respect both reviewers' suggestions and the authors' original vision, while also emphasizing the importance of organization and clarity in presenting revisions. Some improvements could be made by simplifying certain recommendations, providing clearer guidance on seeking editorial approval, and streamlining references. The suggestion to use tables to organize responses to reviewers is excellent. This method is often recommended as it allows for a clear, organized presentation of how each comment was addressed, helping both reviewers and editors to easily follow the changes made.

**RESPONSE**

 We thank the reviewer for this thoughtful suggestion.

 No action is needed.

**COMMENT 7.**

 Revise and improve

 A solid foundation for authors navigating the manuscript revision process is offered in this section. It covers key areas such as documentation, communication, and final review, with a clear emphasis on professionalism and attention to detail. However, I recommend reducing repetitive focus on gratitude (A single, well-crafted expression of thanks may be sufficient).

**RESPONSE**

 We thank the reviewer for this thoughtful suggestion.

 We have reduced the repetitive focus on gratitude.

**COMMENT 8.**

**AI Tool Use**: The mention of AI tools for proofreading is timely, but the section could benefit from a brief discussion on the limitations of these tools. Authors should be cautious, as AI tools may not catch all linguistic nuances or contextual errors. A recommendation to use AI tools as a supplementary measure, rather than a primary one, would be more balanced. The inclusion of a brief discussion on the limitations of AI tools and a more detailed reference to the appendix would also improve the section’s overall effectiveness.

**RESPONSE**

 We acknowledge the timely mention of AI tools for proofreading.

 We have added the following sentence inside the “Review again” section:

 Authors should be cautious, as artificial intelligence tools may not catch all linguistic nuances or contextual errors, which can lead to oversight. Authors are advised to use artificial intelligence tools as a supplementary measure, rather than a primary one. This approach ensures a more balanced and thorough proofreading process. **^4, 38, 41^**

**COMMENT 9.**

 Conclusion

 The authors provide a strong summary of the PRP’s significance for both publishers and authors. The conclusion effectively encapsulates the dual roles of the PRP for both publishers and authors. By highlighting how the PRP not only serves as a quality control mechanism for publishers but also provides authors with valuable feedback, the conclusion underlines the significance of this process in academic publishing.

 I recommend the indexing of this paper after considering the above mentioned comments.

**RESPONSE**

 We thank the reviewer for this excellent feedback.

 No action is needed.

**COMMENT 10.**

 Is the topic of the opinion article discussed accurately in the context of the current literature?

 Yes

 Are all factual statements correct and adequately supported by citations?

 Yes

 Are arguments sufficiently supported by evidence from the published literature?

 Yes

 Are the conclusions drawn balanced and justified on the basis of the presented arguments?

 Yes

**RESPONSE**

 Thank you for your positive feedback and for the 4 “yes”.

# Reviewer report: Reviewer response for version 1

**DOI:** 10.5256/f1000research.169662.r316498

**Published:** 2024-09-05

**Authors:** Abdelghani Maddi

**Affiliations:** 1 CNRS and Sorbonne University, Paris, France

**I. General comment:**

 The manuscript is a well-crafted opinion piece that provides insightful guidance for authors on how to respond effectively to reviewers' comments during the peer review process (PRP). It is particularly valuable for early-career researchers who may be navigating the complexities of peer review for the first time, as well as for more experienced scholars who encounter ambiguous or challenging situations. While the manuscript is commendable for its comprehensive coverage and practical advice, there are several areas where it could benefit from additional clarification and refinement, particularly by addressing some specific issues and minimizing redundancy to enhance readability.

**II. Strengths:**

- **Comprehensive Coverage of PRP Strategies:**
  The manuscript succeeds in presenting a wide array of strategies and practical advice that are relevant to the peer review process. It covers essential aspects of how to respond to reviewer comments, ranging from addressing specific critiques to maintaining a constructive tone throughout the communication. This breadth of coverage provides authors with a robust toolkit to approach various scenarios they may encounter in their scholarly communications.
- **Clear Section Structuring:**
  The logical organization of the manuscript, with well-defined headings and subheadings, greatly aids in its readability. By structuring the content into distinct sections, the authors make it easier for readers to navigate the text and locate the information most relevant to their particular situation. This clarity in organization also helps to highlight the key messages and guidance offered, thereby enhancing the utility of the manuscript as a reference guide.
- **Practical and Applicable Advice:**
  The article excels in offering actionable advice that authors can readily apply when responding to reviewers. For example, the suggestion to break down complex reviewer comments into manageable parts and to address each point methodically helps demystify what can often feel like an overwhelming process.
- **Emphasis on Professionalism and Constructive Dialogue:**
  A key strength of the manuscript is its consistent emphasis on professionalism and the promotion of constructive dialogue. By advocating for a balanced approach that combines firmness in defending one’s scholarly contributions with openness to constructive feedback, the manuscript reinforces the importance of maintaining a respectful and cooperative dynamic between authors and reviewers.

**III.** **Areas for Improvement:**

 Despite these strengths, there are several areas where the manuscript could be further enhanced by incorporating additional insights and clarifying certain points. I offer the following suggestions based on my personal experience as both an author and a reviewer:

- **Addressing Requests for Non-Citation of Specific Authors in the "Ambiguous Situations" Section:**
  It would be beneficial for the manuscript to include guidance on handling situations where reviewers request that specific authors not be cited due to reputational concerns or prior behavior. In such cases, it is vital to determine whether the request pertains to the author as an individual or to the research itself. If the request is directed at the author for personal or ethical reasons, authors should consider contacting the editor privately to discuss the best course of action. This scenario is increasingly relevant in light of the growing prominence of post-publication peer review platforms such as PubPeer, where scholars openly critique the work of their peers. This transparency can sometimes lead to contentious situations, where certain researchers may be effectively "blacklisted" due to their public critiques. Addressing this issue would provide authors with a clearer understanding of how to manage such delicate situations.
- **Incorporating a Practice of Acknowledging Reviewers in the "Express Gratitude Again" Section:**
  The manuscript could be further enriched by suggesting the inclusion of reviewers in the acknowledgements section of the article when their feedback has substantially contributed to improving the manuscript. Beyond thanking reviewers in the response letter, a formal mention in the acknowledgements section demonstrates the authors’ appreciation for the constructive role reviewers play in refining scholarly work. This practice not only fosters goodwill but also acknowledges the often-overlooked labor of peer reviewers who contribute to the advancement of knowledge through their insights.
- **Highlighting the Issue of Plagiarism of Reviewers' Comments:**
  An additional point worth addressing is the problematic practice of directly copying and pasting reviewers' comments into the manuscript. This practice may be viewed as a lack of originality and could raise concerns regarding academic integrity. It is advisable that if reviewers' comments provide valuable suggestions for improvement, these ideas should be paraphrased and integrated into the manuscript in the authors’ own words. An extreme example can be drawn from my own experience, where authors copied nearly a full page of my comments verbatim to create a new "Limitations" section in a paper published by a “gray” publisher. Emphasizing the importance of originality in incorporating reviewer feedback would help maintain the scholarly standards of the work.
- **Reducing Redundancies and Repetition for Greater Clarity:**
  While the manuscript provides many useful insights, there are instances where certain points, such as those concerning professionalism and the manner of responding to reviewers, are repeated multiple times throughout the text. Streamlining these sections by consolidating similar points would help reduce redundancies and improve the overall flow of the manuscript, making it more concise and engaging for the reader.

To sum up, this manuscript is a highly valuable resource for authors seeking to effectively navigate the peer review process. The incorporation of the suggested improvements would further enhance its comprehensiveness and clarity, ensuring that it continues to serve as a practical guide for both new and experienced researchers.

Is the topic of the opinion article discussed accurately in the context of the current literature?

Yes

Are arguments sufficiently supported by evidence from the published literature?

Yes

Are all factual statements correct and adequately supported by citations?

Yes

Are the conclusions drawn balanced and justified on the basis of the presented arguments?

Yes

Reviewer Expertise:

Scientometrics, Research Evaluation, Peer Review, Research Integrity.

I confirm that I have read this submission and believe that I have an appropriate level of expertise to confirm that it is of an acceptable scientific standard.

# Response

**Published:** 2024-09-06

**Authors:** Helmi BEN SAAD

**Affiliations:** Université de Sousse, Tunisia

The authors sincerely thank the reviewer for his thorough and insightful feedback on our manuscript. We have carefully considered each comment and made the necessary revisions to address his concerns. We believe these changes have significantly improved the manuscript.

 Below are our specific responses to the reviewer positive comments/suggestions.

**COMMENT 1**. I. General comment:

 The manuscript is a well-crafted opinion piece that provides insightful guidance for authors on how to respond effectively to reviewers' comments during the peer review process (PRP). It is particularly valuable for early-career researchers who may be navigating the complexities of peer review for the first time, as well as for more experienced scholars who encounter ambiguous or challenging situations. While the manuscript is commendable for its comprehensive coverage and practical advice, there are several areas where it could benefit from additional clarification and refinement, particularly by addressing some specific issues and minimizing redundancy to enhance readability.

**RESPONSE**

 We thank the reviewer for his valuable feedback and for recognizing the potential impact of our manuscript on both early-career and experienced researchers. We appreciate his thoughtful suggestions regarding areas for clarification and the need to minimize redundancy. We agree that enhancing the readability of the paper will strengthen its overall value, and we have refined the manuscript accordingly. By addressing the specific issues highlighted by the reviewer, we aimed to further improve its clarity and utility for all readers. As the reviewer aptly mentioned, together we can achieve more, and we sincerely appreciate the reviewer’ contribution in helping us move towards that goal. We thank the reviewer again for his constructive comments and support.

**COMMENT 2**. II. Strengths:

 *Comprehensive Coverage of PRP Strategies:

 The manuscript succeeds in presenting a wide array of strategies and practical advice that are relevant to the peer review process. It covers essential aspects of how to respond to reviewer comments, ranging from addressing specific critiques to maintaining a constructive tone throughout the communication. This breadth of coverage provides authors with a robust toolkit to approach various scenarios they may encounter in their scholarly communications.

 *Clear Section Structuring:

 The logical organization of the manuscript, with well-defined headings and subheadings, greatly aids in its readability. By structuring the content into distinct sections, the authors make it easier for readers to navigate the text and locate the information most relevant to their particular situation. This clarity in organization also helps to highlight the key messages and guidance offered, thereby enhancing the utility of the manuscript as a reference guide.

 *Practical and Applicable Advice:

 The article excels in offering actionable advice that authors can readily apply when responding to reviewers. For example, the suggestion to break down complex reviewer comments into manageable parts and to address each point methodically helps demystify what can often feel like an overwhelming process.

 *Emphasis on Professionalism and Constructive Dialogue:

 A key strength of the manuscript is its consistent emphasis on professionalism and the promotion of constructive dialogue. By advocating for a balanced approach that combines firmness in defending one’s scholarly contributions with openness to constructive feedback, the manuscript reinforces the importance of maintaining a respectful and cooperative dynamic between authors and reviewers.

**RESPONSE**

 We sincerely thank the reviewer for his positive and thoughtful feedback. We are delighted that the reviewer found our manuscript comprehensive and practical, particularly in its coverage of PRP strategies. It is reassuring to know that the breadth of our approach and the actionable advice provided resonated with the reviewer and that the logical structuring of the sections contributed to its readability and utility as a reference guide. We are also pleased that our emphasis on professionalism and fostering constructive dialogue between authors and reviewers was appreciated, as these are core principles we aim to promote. The recognition by the reviewer of these strengths reinforces our belief in the manuscript's potential to support authors in navigating the complexities of the PRP. We thank the reviewer again for his insightful comments and for recognizing the strengths of our work

**COMMENT 3**. III. Areas for Improvement:

 Despite these strengths, there are several areas where the manuscript could be further enhanced by incorporating additional insights and clarifying certain points. I offer the following suggestions based on my personal experience as both an author and a reviewer:

**3.a. Addressing Requests for Non-Citation of Specific Authors** in the "Ambiguous Situations" Section:

 It would be beneficial for the manuscript to include guidance on handling situations where reviewers request that specific authors not be cited due to reputational concerns or prior behavior. In such cases, it is vital to determine whether the request pertains to the author as an individual or to the research itself. If the request is directed at the author for personal or ethical reasons, authors should consider contacting the editor privately to discuss the best course of action. This scenario is increasingly relevant in light of the growing prominence of post-publication peer review platforms such as PubPeer, where scholars openly critique the work of their peers. This transparency can sometimes lead to contentious situations, where certain researchers may be effectively "blacklisted" due to their public critiques. Addressing this issue would provide authors with a clearer understanding of how to manage such delicate situations.

**3.b. Incorporating a Practice of Acknowledging Reviewers in the "Express Gratitude Again" Section:**

 The manuscript could be further enriched by suggesting the inclusion of reviewers in the acknowledgements section of the article when their feedback has substantially contributed to improving the manuscript. Beyond thanking reviewers in the response letter, a formal mention in the acknowledgements section demonstrates the authors’ appreciation for the constructive role reviewers play in refining scholarly work. This practice not only fosters goodwill but also acknowledges the often-overlooked labor of peer reviewers who contribute to the advancement of knowledge through their insights.

**3.c. Highlighting the Issue of Plagiarism of Reviewers' Comments:**

 An additional point worth addressing is the problematic practice of directly copying and pasting reviewers' comments into the manuscript. This practice may be viewed as a lack of originality and could raise concerns regarding academic integrity. It is advisable that if reviewers' comments provide valuable suggestions for improvement, these ideas should be paraphrased and integrated into the manuscript in the authors’ own words. An extreme example can be drawn from my own experience, where authors copied nearly a full page of my comments verbatim to create a new "Limitations" section in a paper published by a “gray” publisher. Emphasizing the importance of originality in incorporating reviewer feedback would help maintain the scholarly standards of the work.

**3.d. Reducing Redundancies and Repetition for Greater Clarity:**

 While the manuscript provides many useful insights, there are instances where certain points, such as those concerning professionalism and the manner of responding to reviewers, are repeated multiple times throughout the text. Streamlining these sections by consolidating similar points would help reduce redundancies and improve the overall flow of the manuscript, making it more concise and engaging for the reader.

 To sum up, this manuscript is a highly valuable resource for authors seeking to effectively navigate the peer review process. The incorporation of the suggested improvements would further enhance its comprehensiveness and clarity, ensuring that it continues to serve as a practical guide for both new and experienced researchers.

**RESPONSE**

 We thank the reviewer for his valuable feedback and for highlighting the strengths of our manuscript. We greatly appreciate his suggestions for improvement based on his experience as both an author and a reviewer. Almost all the reviewer’ suggestions were considered. We thank again the reviewer for his time and thoughtful review.

**3.a. **We thank the reviewer for his insightful comment and for highlighting the importance of addressing requests for non-citation of specific authors due to reputational concerns or prior behavior. This is indeed a relevant issue in today’s academic environment, particularly with the increasing transparency brought by platforms like *PubPeer*. We agree that providing guidance on how to navigate such delicate situations would be valuable for authors, and we have included this in the “Ambiguous Situations” section. We thank again the reviewer for his thoughtful feedback.

 We have added the following sentence:

 Finally, when reviewers express concerns about citing specific authors due to their reputation or past behavior, it is crucial to evaluate whether the issue is related to the individual or the research itself. If the request is rooted in personal or ethical concerns, authors should consider discreetly contacting the editor for guidance. With the increasing popularity of post-publication peer review platforms like *PubPeer*, *ScienceOpen*, and *PREreview*, such situations may become more common, necessitating careful consideration by authors. **^35^** These steps/approaches are crucial for maintaining transparency and professionalism and the integrity of the PRP, and ensuring fair and ethical treatment of manuscripts.

**3.b. **We thank the reviewer for his thoughtful suggestion regarding the inclusion of reviewers in the acknowledgements section of the manuscript. We agree that acknowledging reviewers, especially when their feedback has significantly contributed to improving the work, is a meaningful way to express gratitude beyond the response letter. This practice not only fosters goodwill but also highlights the important role reviewers’ play in advancing scholarly work. We have incorporated this recommendation into the "Express Gratitude Again" section, as it aligns with our goal of promoting a respectful and transparent peer review process. We have also acknowledged the reviewers of our paper in our acknowledgements section (Please see our revised section: Acknowledgments).

 We have added the following text:

 Finally, including reviewer acknowledgements in the article's “acknowledgements section” can strengthen the manuscript. When reviewers' feedback has substantially improved the work, formally recognizing their contributions demonstrates appreciation beyond the response letter. **^15^** This practice not only cultivates positive relationships but also acknowledges the crucial role of peer reviewers in advancing knowledge. **^15^** By highlighting their efforts, authors recognize the often-underappreciated work that reviewers do to refine scholarly articles. **^15^**

**3.c. **We thank he reviewer for highlighting the issue of directly copying and pasting reviewers' comments into the manuscript. We agree that this practice undermines originality and raises concerns about academic integrity. The reviewer experience underscores the importance of properly paraphrasing and integrating valuable suggestions from reviewers into the manuscript. We addressed this point in our paper to emphasize the importance of maintaining originality while incorporating feedback. In the section “Pay attention to details: Respond to every point raised” we have highlighted the issue of plagiarism of reviewers' comments and we have added the following sentence :

 Finally, to uphold scholarly integrity, it is essential to avoid directly copying and pasting reviewers' comments into the manuscript. ^8^ **^, 28^** Instead, valuable suggestions provided by reviewers should be paraphrased and integrated into the manuscript using the author's own words. ^8^ **^, 28^** This practice not only maintains academic standards but also ensures that the incorporation of feedback reflects originality and thoughtful engagement with the review process. ^8^ **^, 28^** Emphasizing this issue reinforces the commitment to academic integrity, **^29^** and enhances the quality of the work.

**3.d. **We thank the reviewer for his valuable feedback regarding the reduction of redundancies and repetition in the manuscript. We appreciate the reviewer’ observation that streamlining the sections on professionalism and responding to reviewers will enhance clarity and improve the flow of the text. We carefully reviewed and consolidated similar points to eliminate redundancies and ensure a more concise and engaging manuscript. Your suggestions helped us refining the presentation of our insights, making the content more accessible and impactful for readers. We thank the reviewer again for his constructive input.

 To sum up, we have made substantial revisions to the manuscript based on the reviewer’ comments/suggestions. We believe these changes have strengthened the quality and clarity of our work. We are grateful for the reviewers and thank him for his positive evaluation of our manuscript and for acknowledging its value as a resource for navigating the PRP. We appreciate the reviewer’s suggestions for further improvements, which will undoubtedly enhance the manuscript’s comprehensiveness and clarity. We have incorporated all his feedbacks to ensure that the guide remains practical and useful for both new and experienced researchers. We thank again the reviewer for his insightful comments and support.

# Reviewer report: Reviewer response for version 1

**DOI:** 10.5256/f1000research.169662.r316501

**Published:** 2024-08-26

**Authors:** Jeffrey N Love

**Affiliations:** 1 Georgetown University, Washington D.C., USA

Hidouri et al have used their experience and the literature to develop a comprehensive approach to guidelines and strategies for authors when dealing with the publication peer-review process.  From my experience and understanding of the literature, the author’s assessment and approach to issues related to peer-review of a submitted manuscript are accurate and substantiated by appropriate citations from a broad array of fields.  This paper is well written and flows nicely.  Table 1 does an excellent job of providing a clear understanding of the issues that may arise as well as a balanced, evidence-based approach to effectively deal with each when responding to the peer-review process. 

 Traditionally, researchers have learned these lessons by trial and error.  I believe that the approach the authors recommend would streamline the “budding” scholars learning regarding the peer-review process with the potential to lead to successful publications earlier than would occur otherwise without the “hard knocks”.  This is the most comprehensive example of guidelines for responding to the peer review process I have seen.  Since these are generally opinion/commentary/perspective publications, a more rigorous approach to evaluating the available literature appears unlikely at this time. 

 As a guest editor of a Special  Issue in Education with a mentored peer-review program for education fellows, I plan on sharing this paper with our fellows and junior authors.  

 Jeffrey N. Love, MD, MHPE, MSc

 Professor Emeritus, Emergency Medicine

 Georgetown School of Medicine

 CORD Guest Editor

 Western Journal of Emergency Medicine 

 Special Issue in Educational Research & Practice

Is the topic of the opinion article discussed accurately in the context of the current literature?

Yes

Are arguments sufficiently supported by evidence from the published literature?

Yes

Are all factual statements correct and adequately supported by citations?

Yes

Are the conclusions drawn balanced and justified on the basis of the presented arguments?

Yes

Reviewer Expertise:

Education Scholarship, Workplace Based Assessments, Program Evaluation, Mentored Peer-Reviews, Vitality of the Workplace

I confirm that I have read this submission and believe that I have an appropriate level of expertise to confirm that it is of an acceptable scientific standard.

# Response

**Published:** 2024-09-05

**Authors:** Helmi BEN SAAD

**Affiliations:** Université de Sousse, Tunisia

Dear Dr. Jeffrey N. Love,

 Thank you for your thoughtful and insightful review of our paper, *Key Guidelines for Responding to Reviewers.*

 We are delighted that you found our approach comprehensive and beneficial for both experienced researchers and those new to the peer-review process. Your comments, particularly regarding Table 1, reaffirm our belief that clear, evidence-based strategies can enhance an author’s ability to navigate peer-review successfully.

 We agree that these lessons are often learned through trial and error, and our goal in sharing these guidelines is to provide a more efficient pathway for emerging scholars. We are honored that you plan to share our paper with your fellows and junior authors in the context of your Special Issue on Educational Research & Practice. It is gratifying to know that our work may contribute to the learning and development of future researchers.

 Thank you again for your kind words and for your commitment to advancing educational scholarship.

 Sincerely,

 Pr Helmi Ben Saad (MD, PhD), On behalf of the co-authors (Hidouri S., Kamoun H., Salah S., Jellad A., Ben Saad H).
